# Serpiginous Travels: A Case of Cutaneous Larva Migrans

**DOI:** 10.7759/cureus.85942

**Published:** 2025-06-13

**Authors:** Nicole Kandinova, Luis Gonzalez, Natalya Goldshteyn

**Affiliations:** 1 Internal Medicine, New York Presbyterian - Brooklyn Methodist Hospital, New York, USA; 2 Infectious Disease, New York Presbyterian - Brooklyn Methodist Hospital, New York, USA

**Keywords:** ancylostoma species, ascaris lumbricoides, cutaneous larva migrans, helminth infection, parasite infection, soil-transmitted helminth (sth)

## Abstract

Cutaneous larva migrans (CLM) is a term used to describe the migrating linear skin lesions caused by the burrowing of parasitic nematode larvae within the epidermis. It may cause significant discomfort and distress to patients despite being a relatively self-limiting condition. We present a case of a 34-year-old female with no significant past medical history who arrived at our institution with a pruritic rash on the left foot. Previous treatment with antivirals and azoles was ineffective. Interestingly, her travel history revealed a recent trip to Barbados which broadened the differential for parasitic infections. The patient was prescribed a course of ivermectin and discharged. She returned to the Infectious Disease clinic three weeks later for follow-up with near resolution of the rash. In our case, we highlight the importance of obtaining a thorough social history when encountering cutaneous lesions as well as key differences in the treatment of hookworm compared to other parasitic infections.

## Introduction

Hookworm-related cutaneous larva migrans (Hr-CLM) is a clinical syndrome that presents as an erythematous linear cutaneous track. Infection results from contact of human skin with the larvae of dog or cat hookworms, most commonly Ancylostoma braziliense or Ancylostoma caninum. Parasitic eggs are shed from animal feces into the soil or sand where they hatch into larvae. The larvae feed off of soil bacteria and undergo two molts before becoming infective. Upon contact with human skin the larvae use their proteasis to penetrate through the stratum corneum. However, they lack the collagenase needed to penetrate into the basement membrane and invade the dermis as they are able to in their animal hosts. The infective cycle continues in natural hosts by penetration into the dermis and subsequent transport via the lymphatic and venous systems to the lungs where they ascend the bronchial tree to the pharynx and are swallowed [1]. The larvae attach to the small intestinal wall where they mature into adults prior to being excreted in the stool. 

Cases of Hr-CLM are more frequently seen in travelers returning from warmer climates such as the Caribbean, South America, and tropical countries of Southeast Asia where larvae commonly inhabit sandy beaches and gardens. Risk of infection is highest in those individuals who have direct skin contact with contaminated sand or soil. Presentation is most commonly seen in patients returning from travel in tropical regions with an estimated infection rate of 2-3% [2] which likely excludes the many cases that are misdiagnosed or missed due to the disease’s self-limiting nature [3]. Our case describes the clinical course of a young female who presented to our institution with a serpiginous eruption of the foot. We highlight the importance of thorough history-taking to recognize CLM lesions which are commonly diagnosed based on clinical presentation alone. Additionally, treatment is highly effective provided that the appropriate anti-parasitic agent is selected.

## Case presentation

**Figure 1:**
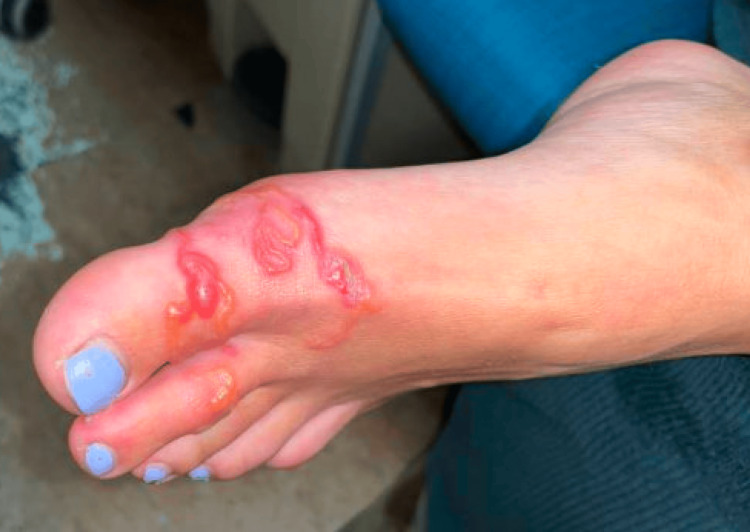
The patient's left foot with a serpiginous track.

A 34-year-old female without a significant past medical history presented to the emergency department for a painful rash on the left foot. She had initially noticed the rash a week prior, and it had since spread rapidly over the skin of the hallux and second digit with associated blistering and erythema. A linear track was observed, as shown in Figure 1. The patient’s primary care doctor had prescribed her valacyclovir and a course of amoxicillin along with topical mupirocin and methylprednisolone. However, the rash progressed. She denied any associated symptoms of fevers, cough, diarrhea, or myalgias. The patient mentioned that the rash initially erupted shortly after returning from a trip to Barbados close to a month prior. She recalled walking on the sand during her trip and feeling a sudden sharp sensation under the left foot. 

The characteristic rash, along with the patient’s travel history, raised suspicion for an *Ancylostoma* infection. She was prescribed ivermectin and discharged from the emergency department. She presented to the Infectious Disease clinic two weeks later for follow-up with near-complete resolution of the rash aside for remaining scab formation.

## Discussion

Cutaneous larva migrans is a clinical diagnosis based on the characteristic dermatological presentation of erythematous, serpiginous, and slightly elevated tracks along the skin. This oftentimes pairs with a history of recent travel to a tropical or subtropical region with subsequent exposure to soil or sand contaminated with animal feces. Lesions usually begin as pruritic erythematous papules at the site of larval penetration, most frequently over the feet, legs, and buttocks. The number of papules correlates with the extent of soil contamination. The larvae then begin to migrate at a rate of several millimeters per day to form severely pruritic, serpiginous, red tracks along the skin within two to six days [4]. They remain within the superficial layers of the skin as they are unable to penetrate the human dermal-epidermal junction. Lesions may extend up to 20 millimeters in length. There is risk of secondary infection as they may become vesiculated or encrusted from frequent scratching. Spontaneous resolution of the lesions within two to eight weeks is likely even without therapy. However, symptoms are subject to recurrence as the larvae may reside in follicles and cause subsequent lesions for up to two years thereafter [5].

Laboratory tests are not required for diagnosis; however, certain parasitology laboratories are available for atypical cases. Biopsy is not recommended as the larvae are usually not histologically identifiable. Dermoscopy has been used to facilitate diagnosis in darker-skinned patients by visualizing the larval bodies that appear translucent and brown alongside empty burrows appearing as dotted vessels. Larvae fragments may also be seen with ultrasonography as hyperechoic subepidermal and intrafollicular structures [6]. 

The main differential diagnosis for the creeping eruption of Ancylostoma is strongyloidiasis. One of the main differences in presentation is the frequent urticaria that accompanies the erythematous “running” subcutaneous larva. These larvae move quickly-approximately 5 to 15 centimeters per hour compared to the 1 to 2 centimeter progression of CLM per day. Contrary to the clinical diagnosis of CLM, strongyloidiasis is usually diagnosed with serological tests that measure serum IgG or Ig4 responses to soluble larvae extracts. While our patient did not have any laboratory tests performed, a more rapidly progressive presentation would warrant serologic investigation for a strongyloides infection [7]. 

Despite the self-limited nature of cutaneous larva migrans infections, antihelminthic therapy is recommended for symptom control and for lowering the rates of bacterial superinfection. First-line treatment is a one-time dose of ivermectin which yields a cure rate of 94 to 100%. Albendazole may be used as a second-line treatment, especially in cases of multiple or extensive lesions requiring a seven-day course [8]. Symptom resolution usually occurs within one week of treatment.

## Conclusions

Cutaneous larva migrans infections are a public health issue across the world. It raises concern for inadequate sanitation and veterinary care in endemic areas. Diagnosis and treatment are frequently delayed by providers outside endemic regions who may mistake the rash for a bacterial infection or environmental sensitivity. This can create opportunities for complications in the form of secondary bacterial superinfection. Thus, this case reinforces the importance of thorough history-taking when evaluating dermatologic complaints, especially in those patients who have traveled to endemic areas. Fortunately, the course of cutaneous larva migrans is usually self-limiting once treated with appropriate antimicrobial coverage. 
